# Clinical Comparison of Airway Stent Placement in Intervention Room and Operating Room

**DOI:** 10.3779/j.issn.1009-3419.2020.104.09

**Published:** 2020-06-20

**Authors:** Ying WANG, Jinming XU, Qi WU, Yuqiong ZHOU, Zhou AN, Wang LV, Jian HU

**Affiliations:** 1 Operating Room, the First Affiliated Hospital, Zhejiang University School of Medicine, Hangzhou 310003, China; 2 Department of Thoracic Surgery, the First Affiliated Hospital, Zhejiang University School of Medicine, Hangzhou 310003, China

**Keywords:** Airway stent placement, Intervention room, Operating room

## Abstract

**Background and objective:**

Airway stent placement is the effective regimen for central airway obstruction (CAO), while its application scenarios varied. This study aimed to make clinical comparison of airway stent placement in the intervention room and operating room.

**Methods:**

Patients underwent airway stent placement between 2014 and 2018 were included in this retrospective case-control study. Clinical performance of airway stent placement in intervention room and operating room were compared.

**Results:**

82 patients were included in this study, including 39 in the intervention room and 43 in the operating room. Patients treated in the intervention room had lower Charlson comorbidity index (CCI) (*P*=0.018) and received less Y-shaped stents (*P* < 0.001). Better clinical response (*P*=0.026), more stents placed (*P* < 0.001) and longer length of stent (*P* < 0.001) were observed in operating room, while there was no significantly statistical difference of stent-related complications and post-stent survival rate between the two groups. Extracorporeal membrane oxygenation (ECMO) supported airway stent placement procedures were performed in the operating room, which provided definitive safety support for high-risk intervention.

**Conclusion:**

Patients with CAO could benefit from the operating room scenario, and airway stent placement in the operating room is more suitable for patients with higher CCI scores and receiving more complicated procedures.

## Introduction

Central airway obstruction (CAO)^[1]^ and tracheoesophageal fistula (TEF)^[2, 3]^ are challenging clinical conditions, which might be caused by both malignant and benign primary diseases. CAO was defined as occlusion of > 50% of the trachea, main bronchi, bronchus intermedius, or a lobar bronchus. It could be classified into extrinsic compression or intrinsic stenosis or the mixed type. With the development of rigid and flexible bronchoscopy^[4]^, airway stent placement becomes the effective and optimal regimen for airway stenosis^[5]^, which could maintain luminal patency for CAO and establish airway integrity for TEF^[6, 7]^, and relieve symptoms rapidly^[8]^.

The application scenarios of airway stent placement varied, including anesthesia approach, airway management, flexible or rigid bronchoscopy types, monitoring equipment, post-anesthesia care facility and emergency treatment support, *etc*.^[9, 10]^. With the development of new technologies, bronchial stenting and laser airway surgery is moving from the operating room to the intervention room^[10]^. While the risk of stent placement and tumor debulking still exists^[11, 12]^, and some researchers even suggested that stenting and laser surgery should not take place outside the operating room^[10]^. The American Society of Anesthesiologists (ASA) suggested that non-operating room anesthetizing locations should have appropriate gas supplies and gas scavenging, sufficient space to accommodate necessary equipment and personnel, expeditious access to the patient, appropriate monitoring equipment, and a post-anesthesia care facility. So far, many studies have focused on the different anesthesia management during airway stent placement^[12, 13]^, while few paid attention on the effect of procedure scenarios. This study aimed to make clinical comparison of airway stent placement in intervention room and operating room, and to propose possible ideas of scenario selection according to patients' status.

## Materials and methods

### Patients

This is a retrospective case-control study. Patients who underwent airway stent placement from January 2014 to January 2018 in the Department of Thoracic Surgery, the First Affiliated Hospital, Zhejiang University School of Medicine were consecutively included. The information was collected from the clinic or electronic medical record system. Patients with CAO caused by benign and malignant airway neoplasm or extrinsic compression, and tracheoesophageal fistula were included, and then they received therapeutic flexible or rigid bronchoscopy in the intervention room or in the operating room. The main symptoms of the patients were dyspnea and dysphagia, and there are many grading systems^[1]^. In this study, dysphagia and dyspnea were graded according to the previous reported methods^[14]^. Charlson comorbidity index (CCI) and Karnofsky performance status (KPS) of the patients were assessed. All patients were included with written informed consent under institutional review board-approved protocols of the First Affiliated Hospital, Zhejiang University School of Medicine.

### Airway stent placement procedure

As reported in our previous study^[6]^, airway stent placement in the intervention room were conducted by therapeutic flexible bronchoscopy under local anesthesia by sprinkling 2% lidocaine via the catheter. And general anesthesia was required for rigid bronchoscopy in the operating room. Stenosis position or fistula was firstly checked, and the guide-wires were inserted and then the stents were released by the bronchoscopy-guided delivery system. Straight or Y-shaped self-expanding covered metallic stents (SEMS) or silicone stents were used in our center. Of note, two cases received extracorporeal membrane oxygenation (ECMO) supported airway tumor debulking and stent placement procedure in the operating room under general anesthesia. All patients were regularly followed up by bronchoscope and telephone calls. The last follow-up time was July 1st, 2018.

### Statistical analysis

The count materials are expressed as number and percentage, and examined by *Chi-square* analysis (*χ*^2^), and the measuring materials are expressed as Mean±SD and examined by Student's *t*-test. All analyses were conducted using the SPSS 22.0 software (IBM SPSS Inc. United States). Statistical significance was set at *P* < 0.05 (All *P* values presented were 2-sided).

## Results

### Clinical efficacy and complications

A total of 82 patients who received stent placement were included in this study. The mean ages were 62.03 and 63.06 years-old for patients in the intervention room and the operating room, respectively. There was no significantly statistical difference for gender, primary diseases, drinking status, smoking status, clinical manifestation, pre-stent therapy, and location of the lesion between the two groups, as shown in Tab 1. Compared with those in the operating room, patients in the intervention room had lower CCI scores (less than 4 points) (*P*=0.018) and underwent less Y-shaped stents (*P* < 0.001).

**1:** Baseline characteristics of stent placement in intervention room and operating room.

Variables	Intervention room (*n*=39)		Operating room (*n*=43)		*P*
	No.	Percent	No.	Percent	
Gender					0.571
Male	28	71.79%	32	74.42%	
Female	10	25.64%	11	25.58%	
Age (yr)	62.03±10.56		63.06±11.50		0.520
Primary disease					0.477
Lung cancer	18	46.15%	15	34.88%	
Esophageal cancer	14	35.90%	21	48.84%	
Others	7	17.95%	7	16.28%	
Drinking					0.313
Yes	19	48.72%	23	53.49%	
No	13	33.33%	17	39.53%	
Unknown	7	17.95%	3	6.98%	
Smoking					0.317
Yes	16	41.03%	20	46.51%	
No	16	41.03%	20	46.51%	
Unknown	7	17.95%	3	6.98%	
Charlson comorbidity index					0.018
< 4	18	46.15%	13	30.23%	
≥5	14	35.90%	28	65.12%	
Unknown	7	17.95%	2	4.65%	
Clinical manifestation					0.815
Obstruction#	27	69.23%	28	65.12%	
Fistula	12	30.77%	15	34.88%	
Pre-stent therapy					1.000
Surgery	18	46.15%	19	44.19%	
Others*	21	53.85%	24	55.81%	
Location					0.066
Above the carina	21	53.85%	32	74.42%	
Below the carina	18	46.15%	11	25.58%	
Y-shaped stent					< 0.001
No	34	87.18%	9	20.93%	
Yes	5	12.82%	34	79.07%	
*: Including chemotherapy, radiotherapy and palliative treatment; #: caused by airway neoplasm or extrinsic compression.					

The clinical efficacy was assessed by rapid clinical response, post-stent complications and survival status. Both groups had significant KPS improvement after stent placement, as shown in Fig 1. Patients in the operating room had better clinical response (*P*=0.026), more stents placed (*P* < 0.001), and longer stents placed (*P* < 0.001), as shown in Tab 2. There was no significant difference of post-stent complications and stent placement time between the two groups. The detailed information of post-stent complications was shown in Tab 3. The percentages of tumor debulking and balloon dilatation procedure were also compared between the intervention room and operating room, and there was a trend that more companion procedures of airway stent placement were conducted in the operating room (*P*=0.051).

**1:**
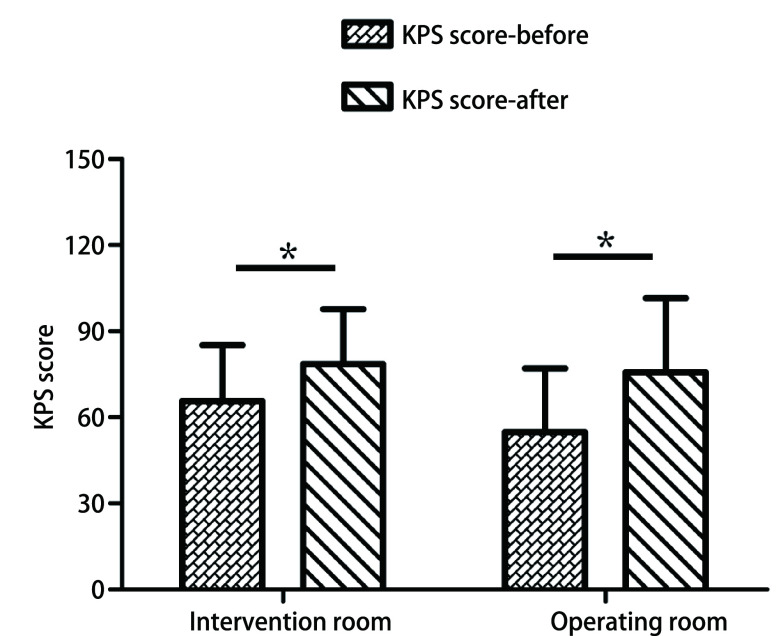
KPS score change for patients before and after stent placement in the intervention room and the operating room. *: *P* < 0.05. KPS: Karnofsky performance status.

**2:** Clinical assessment of stent placement in intervention room and operating room

Variables	Intervention room (*n*=39)		Operating room (*n*=43)		*P*
	No.	Percent	No.	Percent	
Clinical response*					0.026
No	25	64.10%	16	37.21%	
Yes	14	35.90%	27	62.79%	
Number of stent					< 0.001
1	38	97.44%	26	60.47%	
2	1	2.56%	16	37.21%	
3	0	0.00%	1	2.33%	
Tumor debulking and balloon dilatation					0.051
No	32	82.05%	26	60.47%	
Yes	7	17.95%	17	39.53%	
Complication					0.278
No	16	41.03%	23	53.49%	
Yes	23	58.97%	20	46.51%	
Length of stent (mm)	43.08±21.68		80.53±38.21		< 0.001
Stent placement time (min)	41.97±38.54		68.62±73.92		0.061
Survival					0.503
No	14	35.90%	19	44.19%	
Yes	25	64.10%	24	55.81%	
*: Clinical response of stent placement was assessed by the symptom improvement grade. Dysphagia was graded on a 5-point scale: no dysphagia (0), able to eat soft food (I), pureed food (II), liquids only (III), and not even liquids (IV). Dyspnea was graded on a 4-point scale: none (0), slight (I), severe (II), and stridor (III). Any grade improvement was defined as positive clinical response.					

**3:** Detailed information of complications after stent placement in intervention room and operating room

Variables	Intervention room (*n*=39)		Operating room (*n*=43)		*P*
	No.	Percent	No.	Percent	
Vocal cord paralysis					0.665
No	36	92.31%	41	95.35%	
Yes	3	7.69%	2	4.65%	
Pulmonary infection					
No	37	94.87%	41	95.35%	1.000
Yes	2	5.13%	2	4.65%	
Hemorrhage					0.617
No	38	97.44%	40	93.02%	
Yes	1	2.56%	3	6.98%	
Restenosis					0.749
No	33	84.62%	38	88.37%	
Yes	6	15.38%	5	11.63%	
Pneumothorax					1.000
No	39	100.00%	41	95.35%	
Yes	0	0.00%	1	2.33%	
Fistula					0.715
No	37	94.87%	41	95.35%	
Yes	2	5.13%	2	4.65%	
Granulation					0.910
No	35	89.74%	32	74.42%	
Yes	4	10.26%	11	25.58%	
Poor expanding					1.000
No	39	100.00%	42	97.67%	
Yes	0	0.00%	1	2.33%	
Atelectasis					0.223
No	37	94.87%	43	100.00%	
Yes	2	5.13%	0	0.00%	
Stent migration					0.715
No	36	92.31%	38	88.37%	
Yes	3	7.69%	5	11.63%	
Stent replacement					0.241
No	30	76.92%	38	88.37%	
Yes	9	23.08%	5	11.63%	
Mucous plugging					1.000
No	34	87.18%	37	86.05%	
Yes	5	12.82%	6	13.95%	

### ECMO supported airway debulking procedure and stent placement in the operating room

A 57-year old female with poorly differentiated squamous cell carcinoma of the left lower lobe was admitted to the hospital for dyspnea, cough and blood in sputum. She was diagnosed as cT2N2M1b (stage IV) lung cancer with metastasis in the adrenal gland 4 years ago, and received 5 times chemotherapy of gemcitabine plus cisplatin until Sep 12, 2015. The central type carcinoma caused obstruction and inflammation of the left lower lobe. The pre-operation CT scans suggested that the tumor had invaded the left main bronchus (Fig 2A and Fig 2B), and there was a trend for the tumor to further invade the right main bronchus with the disease progress, which could cause life-threatening death. Under this circumstance, an ECMO-supported airway tumor excavation by rigid bronchoscopy was performed. The left main bronchus was excavated to 4.5 cm, but the bronchial opening of left upper and lower lobe still could not be seen. The ECMO was removed one day after surgery, and the post-operation bronchoscopy was shown in Fig 3A, Fig 3B, Fig 3C. The patient underwent airway stent placement under general anesthesia with endotracheal intubation 20 days later. Two Y-shaped stents were placed in the left and right main bronchus, the right middle and upper bronchus, respectively (Fig 3D, Fig 3E, Fig 3F) and there was much phlegm in the airway. The post-stent CT scans suggested the main airway and right bronchus were unobstructed (Fig 2C and Fig 2D). This patient received several times of sputum aspiration by bronchoscopy and was still alive until the last follow-up time.

**2:**
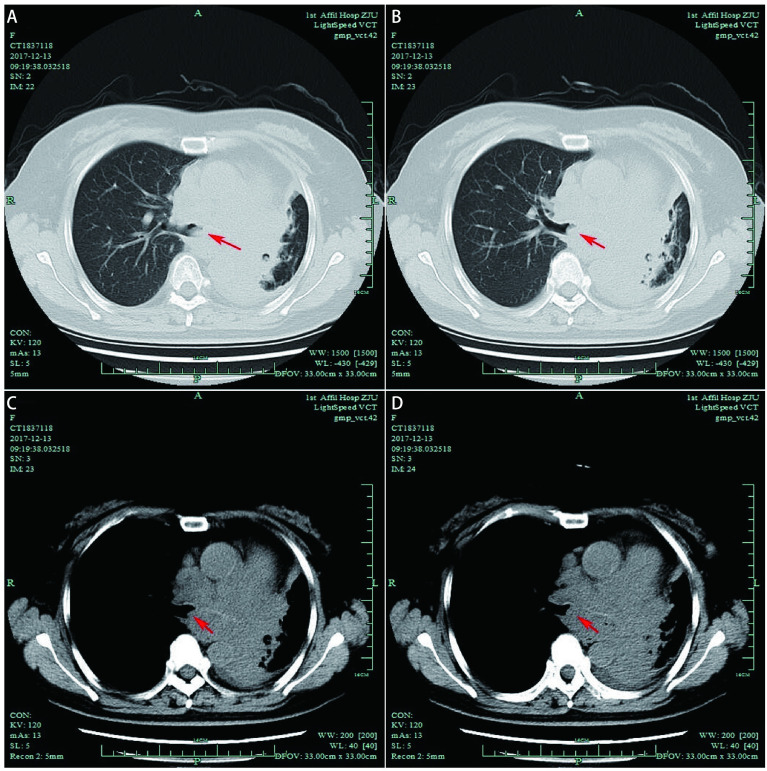
Case 1. Pre-stent lung window (A) and mediastinal widow (B) of the ECMO supported stenting case, and the red arrow suggested the location of tumor. Post-stent lung window (C) and mediastinal widow (D) of the case, and the red arrow suggested the stent position.

**3:**
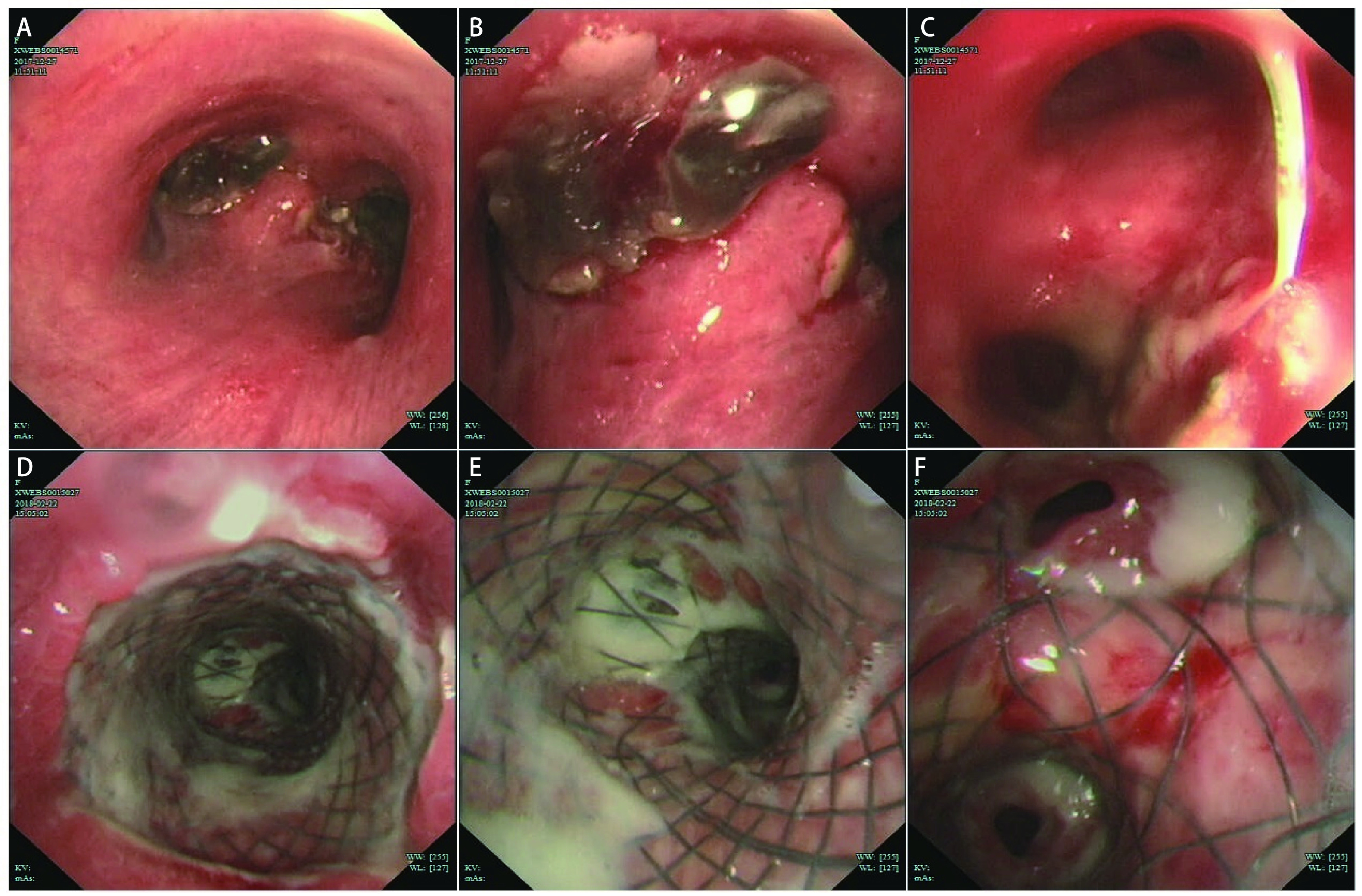
Bronchoscopy of pre-stent (A, B and C) and post-stent (D, E and F) images. A: Tumor invading carina from the view of main trachea. B: Amplified view of A. Left main bronchus was blocked by tumor. C: The right bronchus was also involved by the tumor. D: Stent was placed in the trachea. E: Stent was placed above the carina to prevent obstruction of main trachea. F: The Y-shaped stent was also placed in right trachea to prevent obstruction of right trachea.

There was another case benefited from the ECMO supported emergency CAO relieving by tumor debulking in the operating room. A 54-year old man was admitted to our department for cough, blood in sputum and dyspnea and was diagnosed as squamous cell carcinoma of the trachea. As shown in Fig 4A and Fig 4B, the main trachea was obstructed and the enlarged subcarina lymph nodes compressed the left and right main bronchus. The patient suddenly occurred decline of partial pressure of oxygen and severe dyspnea, and ECMO supported trachea neoplasm debulking and airway remodeling were conducted after the evaluation of the anesthesiologist. The patient was in a stable condition after the procedure, and then the Y-shaped stents were placed by rigid bronchoscopy under general anesthesia 8 d later, as shown in Fig 4C and Fig 4D. The patients was still alive until the last follow-up time.

**4:**
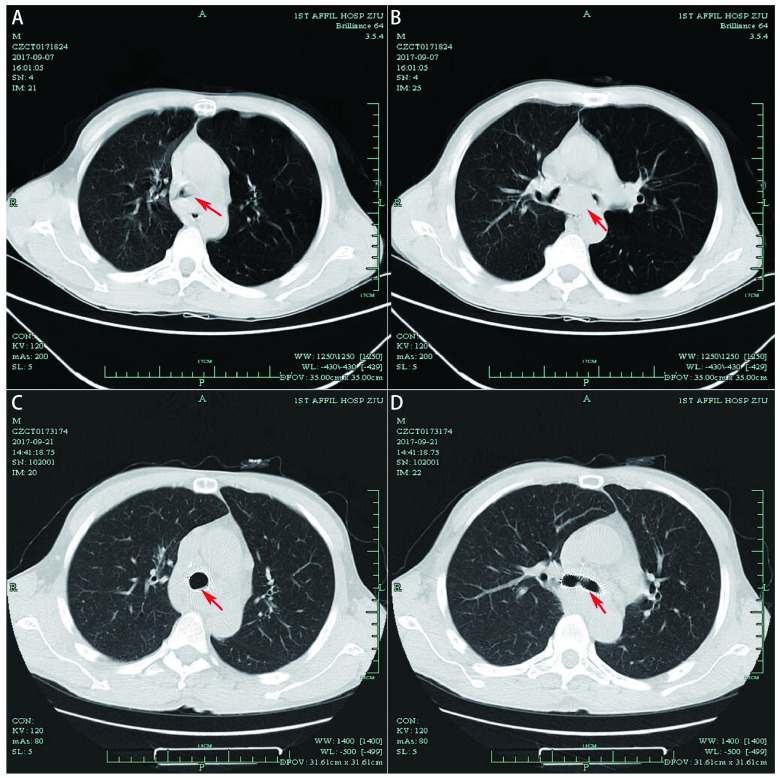
Case 2. Pre-stent lung window (A) and mediastinal widow (B) of the ECMO supported stenting case, and the red arrow suggested the location of tumor. Post-stent lung window (C) and mediastinal widow (D) of the case, and the red arrow suggested the stent position.

## Discussion

The current study made clinical comparison of airway stent placement in different application scenarios. Clinical performance, post-stent complications and survival of airway stent placement in the intervention room and the operating room were compared. Our study found that more complicated procedures were performed in the operating room, including more stents, more Y-shaped stents and stents with longer length. On the other hand, patients underwent airway stent placement in the operating room had higher CCI scores (≥5 points). An ECMO supported stent placement and tumor debulking procedure was performed in the operating room, which suggested that the operating room could provide definitive security support.

For patients with CAO and TEF, airway stent placement could timely provide symptom palliation and improve survival and quality of life^[15, 16]^. The application scenarios of airway stent placement, stent types, anesthesia methods, bronchoscopy types, operating doctor's experience and nursing care varied, which could inevitably affect the safety and efficacy. Some researchers have suggested that patients' safety should always be a primary consideration, and bronchial stenting and laser airway surgery should not take place outside the operating room^[10]^. Operating room could provide adequate safety support for high risk intervention procedure, such as necessary equipment and personnel. The high-tech platform including ECMO, monitoring equipment, airway management equipment, rescue drugs and equipment for emergencies and the multidisciplinary team consisting of anesthesiologists, thoracic surgeons, and intervention experts could ensure patient's safety made further treatment possible^[17]^. Airway stent placement is always risky, and some studies have suggested that it should only be performed at specialized centers by experienced physicians^[18]^.

Stent-related complications are inevitable, and many studies have reported these complications^[9, 18-21]^. In this study, post-stent complications were analyzed, including pulmonary infection, hemorrhage, restenosis, pneumothorax, fistula, granulation, poor expanding, atelectasis, stent migration and replacement, and mucous plugging. Airway stent placement in the operating room suggested lower rate of total complications than in the intervention room. Besides, more complicated procedures were conducted in the operating room, including two cases of ECMO supported tumor debulking and airway stent placement. Respiratory support with ECMO during stent placement have been reported in several studies^[22, 23]^, and most cases receive ECMO in the intensive care unit. Although potentially life-threatening complications exist, such as bleeding, hemolysis, air leakage, and thrombosis^[24]^, ECMO could provide sufficient oxygenation during airway interventional procedures and definitive airway security. It has been reported that even the rapid bedside ECMO could save patients' life in experienced centers, and the complications could be managed by an experienced ECMO team^[25]^. In our study, the ECMO supported tumor debulking procedures were conducted in the operating room, which further ensured the security for high-risk patients and emergencies.

The material quality and shapes of airway stents varied. In this study, the main types were straight or Y-shaped SEMS or silicone stents. The Food and Drug Administration have called for restraint of metallic stents for benign airway disorders in 2005^[26]^, considering the effects of complications on the long-term survival of patients with benign diseases. However, many studies further reported the safety and efficacy of SEMS in benign^[27]^ and malignant^[17, 19]^ airway stenosis. Ayub *et al*. suggested that silicone stents have shown low complication rates and can ultimately be removed and should be first choice for patients with benign stenosis^[18]^. For malignant CAO patients with shorter life expectancy, both the SEMS and silicone stents seem to have similar efficacy and safety profile^[28]^. Matsumoto *et al*. even suggested that the combination stenting with silicone and metallic stents is a safe and beneficial procedure for patients with extended malignant airway stenosis^[29]^. More well-designed studies with large sample volume are warranted to further investigate this issue.

Careful patient selection is of outmost importance for a good outcome after airway stenting^[4]^. So far, there was no systematic selection process for higher risk CAO patients undergoing airway stent placement and this is the first time to compare different procedure scenarios. The patients' CCI scores were higher in the operating room, and they had lower rates of post-stent complications, which indicated that higher risk patients could benefit from the operating room scenarios. Extremely high risk patients and emergency cases could benefit from the ECMO technique. Thus, preoperative evaluation and standard patient selection were important, for the consideration of both efficacy and security. This study proposed the idea that patients of different risk intervals should be evaluated and treated under standardized and personalized protocols, and more studies could further investigate this filed in the future.

In conclusion, patients with benign or malignant CAO could benefit from the operating room scenario, and airway stent placement in the operating room might be more suitable for patients with higher CCI scores and receiving more complicated procedures (more, longer, and Y-shaped stents).

Patients with benign or malignant CAO could benefit from the operating room scenario, and airway stent placement in the operating room might be more suitable for patients with higher CCI scores and receiving more complicated procedures (more, longer, and Y-shaped stents).

## Acknowledgements

This work was funded by National Key R & D Program of China (to Jian Hu) (No.2017YFC0113500), Traditional Chinese Medicine Research Fund Program of Zhejiang Province (to Ying WANG) (No.2017ZA084), Medical and health science and technology projects of Zhejiang Province (to Ying WANG)(No.2018ZH010 and No.2019KY069), and General scientific research projects of Zhejiang Provincial Department of Education(to Ying WANG) (No. Y201839014)

## Author contribution

Wang Y and Hu J conceived and designed the study. Wang Y and Xu JM contributed to data analysis and editing the manuscript. Wu Q and Zhou YQ contributed to data acquisition, statistical analysis and interpretation of the data. An Z contributed to clinical stent placement. Lv W contributed to the revision of the manuscript. All authors read and approved the final manuscript as submitted.
